# Authors' reply: Re: Yang *et al*. The road to therapy for myeloid sarcoma: navigating the complexities of subclonal MAPK/ERK mutations and clonal evolution

**DOI:** 10.1002/2056-4538.70064

**Published:** 2025-11-17

**Authors:** Dominik Nann, Falko Fend

**Affiliations:** ^1^ Institute of Pathology and Neuropathology University Hospital Tuebingen and Comprehensive Cancer Center South West Tuebingen Germany

Reply to Yang *et al*.

We thank the authors for their interest in our recent paper ‘Myeloid sarcoma shows a high frequency of mutations activating the MAPK/ERK pathway and association with clonal hematopoiesis’ [1], which appeared in the September issue of *The Journal of Pathology, Clinical Research*. In their letter to the Editor, Dr. Yang and his colleagues raise some important issues, which point the way for further studies on myeloid sarcoma (MS) and its development [2]. These include subclonal mutations in the MAPK pathway, the association with clonal hematopoiesis and the importance of the microenvironment.

Nevertheless, we want to clarify a few points raised by Dr. Yang and colleagues concerning our results. They state that the frequent subclonal nature of many MAPK/ERK mutations in our MS samples suggests they may not be the universal founding oncogenic event. Although we agree with this statement in principle, since activating mutations of *NRAS, KRAS* and/or *BRAF* were observed in only 53% of our MS cases and other acquired mutations, namely *NPM1*, mutated in 38% of cases, are alternative candidate drivers, we want to point out that *NRAS/KRAS* mutations constitute minor, subclonal events in only 2/18 cases. In case 27, the bone marrow showed a *KRAS* mutation with 7% variant allele frequency (VAF), which was present at 2% and 3% VAF, respectively, in the two MS from this patient. In case 31, the *NRAS* mutation was present in the bone marrow at 28% VAF and at only 0.4% VAF in the MS. In these cases, the MAPK/ERK mutations most likely represent a subclone and are not responsible for MS development. In the remaining 15/18 cases with *NRAS/KRAS* mutations; however, these showed a high VAF (mean 41.7%, range 19–92%). In the cases with available pre‐transplant bone marrow (BM) biopsy, the majority of *NRAS/KRAS* mutations were acquired in MS or showed a significant increase in VAF in the MS in comparison to the BM biopsy. In some cases with mutations in other genes with higher VAF, this is indicative of a bi‐allelic event rather than subclonality of the *RAS* mutation, as for example in case 33 with a *TP53* mutation with 83% VAF and the *EZH2* mutation in case 18 with 89% (scrotal) and 99% (penis) VAF, respectively, in two different locations. In summary, the high VAFs and the frequent *de novo* acquisition of *NRAS/KRAS* mutations in many MS cases, together with the clear evidence for upregulation of the MAPK/ERK signaling cascades (supplementary material, Figure S3 in our paper [1]) detected by comparative gene expression profiling clearly point to a role in MS development, although functional studies will be necessary to corroborate these findings. Of interest is case 10, which showed acquired identical mutations of *BRAF* and *KRAS* in both MS samples, but not in the BM. Given the fact that both mutations affect the same pathway, it is tempting to assume the presence of two competing subclones present in both MS manifestations.

The second issue raised by Dr. Yang and co‐workers is the association of MS with clonal hematopoiesis (CH). They argue that the relationship between CH and MS remains largely inferential and would require different techniques for confirmation. Although single‐cell sequencing allows a more stringent delineation of clonal evolution, the presence of identical CH‐type mutations (*TET2, DNMT3A, ASXL1*, among others) shared between bone marrow and MS in all five cases with clonal hematopoiesis, combined with high VAFs in the MS, effectively rules out contamination by accompanying inflammatory CH cells not belonging to the MS clone. In our opinion, this provides strong evidence for a clonal relationship with CH and, together with the acquisition of private mutations in the MS known to be drivers for leukemic transformation, indicates a role for the MAPK/ERK pathway and other activating events, such as the acquisition of *NPM1* and possibly *EZH2* mutations in the pathogenesis of CH‐associated MS. Nevertheless, we agree with Dr. Yang and co‐workers that branched evolution, a common feature in many malignant disorders including leukemias, as well as the microenvironment, is likely to represent influential factors in MS development. We also believe that CH‐associated MS represents a different disease from translocation‐driven MS in young patients, although the number of such cases with evaluable BM biopsy in our series was too small for any conclusions.

As discussed by the authors, their hypothesis that MS actively remodels the tumor microenvironment (TME) and that CH‐associated and true *de novo* MS might differ in their TME will require different methods of investigation to provide insights into the crosstalk between malignant cells and surrounding immune cell populations. Of note, gene set enrichment analysis of our comparative transcriptional data of MS revealed extracellular matrix organization, peptidase regulator activity and protease and chemokine receptor binding as important discriminating pathways, supporting an active role of malignant cells in shaping the stromal niche of MS. A detailed analysis of the TME of MS, however, was beyond the scope of our work.

In summary, Dr. Yang and colleagues have developed important ideas based on our findings, and we are thankful for their appreciation of our work. Future studies using advanced technologies for dissecting clonal trajectories and the TME of MS will advance our knowledge of this prognostically frequent dismal disease.

## Author contributions statement

FF wrote the first draft. FF and DN reviewed and critiqued the manuscript, and gave final approval.
